# Evaluating the effects of metronidazole, metformin, and onion extract on GAPDH activity and nitric oxide production in *Blastocystis* sp. cultures: a biochemical and therapeutic approach

**DOI:** 10.1186/s12906-026-05605-x

**Published:** 2026-10-12

**Authors:** Gehad A. Basuony, Alshaimaa M. R. Hamed, Amal M. Mahfoz, Abdel Wahab M. Mahmoud, Hala Ramadan, Magda S. A. Abdeltawab

**Affiliations:** 1 Department of Medical Parasitology, Faculty of Medicine, Cairo University, Cairo, Egypt; 2 Egyptian Drug Authority, Cairo, Egypt; 3 Saudi Toxicology Society, Department of Pharmacology and Toxicology, College of Medicine, Umm Al Qura University, Makkah, Saudi Arabia; 4 Plant physiology Department, Faculty of Agriculture, Cairo University, Giza, 12613 Egypt; 5 Department of Internal Medicine, Faculty of Medicine, Cairo University, Cairo, Egypt

**Keywords:** *Blastocystis sp*., Metronidazole, Metformin, Onion extract, GAPDH, Nitric oxide

## Abstract

**Background:**

*Blastocystis* sp. is a highly prevalent anaerobic intestinal protozoan increasingly associated with gastrointestinal disorders. Its survival within the gut microenvironment depends on metabolic pathways such as glycolysis, where glyceraldehyde-3-phosphate dehydrogenase (GAPDH) plays a central role, alongside interactions with nitric oxide (NO) derived from host defenses and surrounding microbiota. The emergence of metronidazole resistance necessitates exploration of alternative therapeutic strategies.

**Objective:**

To evaluate the in vitro effects of metronidazole, metformin, and *Allium cepa* (onion) extract on *Blastocystis* sp. growth, and GAPDH activity and NO levels within the xenic culture medium.

**Methods:**

Twenty-one *Blastocystis*-positive stool samples were cultured in Jones’ medium under xenic conditions. Cultures were treated with graded concentrations of metronidazole, metformin, and onion extract. Parasite counts were assessed microscopically, while GAPDH activity and NO levels were quantified using gas chromatography–mass spectrometry (GC–MS)-based analysis.

**Results:**

All tested agents significantly reduced *Blastocystis* counts compared with untreated controls (*p* < 0.05), with metronidazole demonstrating the greatest antiparasitic effect. Significant reductions in NO levels and GAPDH activity were observed across all treatment groups (*p* < 0.001). Metformin (1000 µg/mL) showed the strongest inhibition of GAPDH activity, whereas metronidazole (500 µg/mL) resulted in the lowest NO levels. Correlation analysis revealed a significant negative association between NO and GAPDH in metronidazole-treated cultures, while a positive correlation was observed with metformin.

**Conclusion:**

Metronidazole, metformin, and onion extract exhibit significant anti-*Blastocystis* activity, accompanied by modulation of key metabolic and nitrosative pathways within the microbial milieu supporting the growth of *Blastocystis* sp. Targeting GAPDH and NO-related mechanisms represents a promising therapeutic approach, particularly in the context of emerging drug resistance and co-colonization with other gut microbiota.

**Supplementary Information:**

The online version contains supplementary material available at https://doi.org/10.1186/s12906-026-05605-x.

## Introduction

*Blastocystis* sp. is one of the most prevalent anaerobic protozoans inhabiting the human gastrointestinal tract, with prevalence rates reaching up to 50% in developing countries. Reports on the association of *Blastocystis* with gastrointestinal manifestations have triggered investigations into its underlying pathogenic mechanisms, which remain poorly understood [1]. The potential pathogenicity of *Blastocystis* is closely linked to its metabolic adaptability. Due to the absence of classical mitochondria, the organism relies primarily on glycolysis for energy production, supported by mitochondrion-like organelles adapted to anaerobic conditions [2, 3]. A key enzyme in this pathway is glyceraldehyde-3-phosphate dehydrogenase (GAPDH), which not only catalyzes a critical step in glycolysis, but also exhibits “moonlighting” functions, including roles in host-parasite interactions, immune evasion, and resistance to oxidative stress [2, 4]. GAPDH has been identified in several gut microbes, including probiotic bacteria, where it was found to play an anti-allergic role through immune modulation and regenerate disrupted tight junctions [5, 6]. The dependence of parasites on glycolysis for energy production renders them more vulnerable to GAPDH inhibitors than their human hosts, which promotes GAPDH as a valuable antiparasitic drug target. The antiprotozoal activity of GAPDH inhibitors has been demonstrated against *Trypanosomes*, *Toxoplasma gondii*, *Plasmodium* spp., and *Leishmania* spp [7]. GAPDH inhibitors were also investigated for their potential therapeutic benefit against helminths such as *Schistosoma mansoni*, by identifying agents that disrupt the GAPDH-triosephosphate isomerase (TPI) complex thereby interfering with GAPDH functions [8]. GAPDH inhibition can also be induced by nitric oxide (NO) through *S*- nitrosylation, thus impeding its glycolytic activity. Moreover, the *S*-nitrosylated form of GAPDH contributes to NO-induced apoptotic cascades, resulting in the activation of p53 [9]. NO constitutes an essential component of host innate immunity, exerting cytotoxic effects against a wide range of pathogens, including *Blastocystis* sp. However, certain subtypes have been shown to modulate host NO responses, enhancing their survival and persistence within the intestinal environment. This interplay highlights the importance of nitrosative stress in the pathophysiology of *Blastocystis* infection [10].

Metronidazole remains the most used antiparasitic agent against *Blastocystis*; however, increasing reports of resistance and treatment failure have raised concerns regarding its efficacy [11]. This has prompted interest in alternative or adjunctive therapeutic approaches. Metformin, a widely used antidiabetic agent, has demonstrated antimicrobial and immunomodulatory effects, including modulation of nitric oxide pathways [12–14]. Similarly, plant-derived compounds such as *Allium cepa* possess bioactive constituents with documented antiparasitic and antioxidant properties [15–17].

Accordingly, this study evaluated the in vitro effects of metronidazole, metformin, and *Allium cepa* extract on *Blastocystis* sp., with a focus on parasite viability, GAPDH activity, and NO levels within a xenic culture milieu. By assessing parasitological and biochemical changes in response to the selected test agents, this work provides preliminary insights into drug-induced *Blastocystis*-culture dynamics in the presence of co-existing intestinal microbiota, to be validated by future mechanistic studies employing axenic cultures or parasite-specific enzymatic assays.

## Materials and methods

### Sample collection and inclusion criteria

Twenty-one *Blastocystis*-positive stool samples were collected from the Diagnostic and Research Unit of Parasitology (DRUP), Faculty of Medicine, Cairo University. A two-step selection process was utilized for this study, where stool specimens were collected via convenience sampling from outpatients presenting with gastrointestinal manifestations, including abdominal pain, diarrhea, nausea, and flatulence [18], and referred for stool analysis, was performed. This was followed by a purposive sampling strategy, where only 21 positive samples were included in the study. All samples were collected in sterile plastic containers and subjected to routine macroscopic and microscopic examinations. Parasite detection was confirmed using direct wet-mount microscopy and the formalin-ethyl acetate concentration technique to definitively identify *Blastocystis*-positive specimens.

Since the primary aim of this study was to investigate the in vitro anti-parasitic and metabolic effects of the tested agents, these 21 isolates were treated strictly as independent biological replicates to initiate parasite propagation, rather than to correlate findings with host demographics or definitive clinical diagnoses (such as irritable bowel syndrome). Exclusion criteria included a history of inflammatory bowel disease, gastrointestinal malignancy, chronic symptoms exceeding six months, or the recent use of antibiotics, proton pump inhibitors, or non-steroidal anti-inflammatory drugs (NSAIDs).

### *Blastocystis* culture and isolation

Jones’ medium was prepared and supplemented with 10% horse serum and 0.1% antibiotic solution containing penicillin G and streptomycin [19]. The pH of the medium was adjusted to 7.0 using Na₂HPO₄, KH₂PO₄, and NaCl before sterilization by autoclaving.

Stool samples positive for *Blastocystis* by microscopic examination were mixed with normal saline and filtered through sterile gauze into sterile centrifuge tubes. The filtrates were centrifuged at 2,000 rpm for 10 min, and the supernatants were discarded. This washing procedure was repeated until the supernatants became clear. The resulting pellet containing *Blastocystis* cells was then aseptically inoculated into labeled culture tubes containing the prepared Jones’ medium using a sterile glass rod. The cultures were incubated at 37 °C for 48–72 h.

### Assessment of the anti-*Blastocystis *effects of the tested agents

#### Rationale for choosing the selected concentrations of the test agents

The efficacy of metronidazole, metformin, and onion extract were tested on *Blastocystis*-positive stool cultures: Metronidazole was tested at 3 different concentrations: 100, 500, 1000 µg/mL, where the of 100 µg/mL concentration was adopted from Yakoob et al. [20], and the 1000 µg/mL concentration was adapted from Roberts et al. [21]. In addition, we have opted to test the effect of an intermediary concentration of 500 µg/mL to bridge the gap between both doses. The dosage choice for metformin and *Allium cepa* extract was particularly challenging, due to lack of satisfactory pharmaceutical and pharmacological literature reports on the effect of these agents on *Blastocystis* sp. in vitro. We have therefore conducted a preparatory pilot study using different drug concentrations, guided by similar studies on in vitro effects of these agents. These studies include Eldeek et al. [22], who investigated the effect of different concentrations of *Allium cepa* on *Allovahlkampfia spelaea* (a free-living amoeba) in vitro. Following preliminary viability trials on *Blastocystis*, concentrations of 500, 1000, and 2000 µg/mL were selected. As for metformin, we have tried different concentrations in a preliminary pilot trial guided by a study conducted by Raafat et al. [23], who tested the in vitro effect of free versus nano-encapsulated metformin on HEP-2 cells, where free metformin was found to induce functional effects on cultured cells at doses > 500 µg/mL.

For the evaluation of GAPDH activity and NO levels, the specific drug concentrations were chosen based on the functional anti-parasitic performance of the drugs, where metronidazole was used in its median concentration of 500 µg/mL to represent a therapeutic baseline for comparing metabolic effects. As for metformin and onion extract, only the lowest onion concentration of 500 µg/mL was excluded because it failed to produce a statistically significant antiparasitic effect compared to the untreated control.

#### Preparation of agents

Metronidazole tablets (Flagyl; Sanofi-Aventis, Suisse, Vernier) were pulverized and dissolved in 10% ethanol-phosphate-buffered saline (PBS) to achieve a stock concentration of 5 mg/mL (5000 µg/mL). Metformin tablets (Merck, Germany) were prepared similarly using the identical 10% ethanol PB vehicle to maintain solvent consistency across all drug preparations. To isolate the active compounds from insoluble binders and stabilizers, both drug mixtures were centrifuged at 3000 rpm for 15 min to precipitate solid fillers. This was followed by filtration through a 0.22 μm syringe filter.

The resulting supernatants were diluted with sterile PBS to prepare intermediate working stocks:

- For the 100 µg/mL treatment (metronidazole): Diluted 1:10 in PBS to prepare an intermediate stock of 500 µg/mL.
- For the 500 µg/mL treatments (metronidazole and metformin): Diluted 1:2 in PBS to prepare an intermediate stock of 2500 µg/mL.
- For the 1000 µg/mL treatments (metronidazole and metformin): The undiluted 5000 µg/mL stock was used directly.

As for the botanical preparation, the cultivated onion (*Allium cepa*, cultivar Giza White) used in this study was harvested directly from the experimental farm of the Faculty of Agriculture, Cairo University, Giza, Egypt. Formal taxonomic identification and preparation of the plant material were performed by co-author Dr Abdel Wahab M Mahmoud, Associate Professor of Plant physiology, Faculty of Agriculture, Cairo University. As this study utilized a standard, non-endangered, cultivated agricultural crop harvested from university experimental land, no wild collection permits were required, and no herbarium voucher specimens were deposited. All plant handling and experimental procedures complied with institutional, national, and international guidelines. Intact fresh onion (*Allium cepa*, ~ 75 g) was minced and ground using a pestle and mortar and passed through a 70–80 mesh sieve to obtain uniform particles. The resulting onion powder was macerated in a solvent mixture of ethanol, methanol (95% v/v, 1:1 ratio), and 100 mL of distilled water, and kept under vigorous magnetic stirring for 48 h. The mixture was then filtered through Whatman No. 1 filter paper to remove bulk particulate matter. The liquid filtrate was concentrated under reduced pressure using a rotary evaporator at low temperature for 2 h, centrifuged at 1200 rpm for 30 min, cooled in an ice bath, and subsequently lyophilized to yield the final purified dry extract [24].

To prepare the onion extract treatment concentrations:

The lyophilized *Allium cepa* powder was reconstituted in sterile physiological PBS to prepare a highly concentrated stock solution of 10 mg/mL (10,000 µg/mL). This stock was diluted with sterile PBS to create intermediate working stocks of:

- 2500 µg/mL (diluted 1:4 in PBS; to achieve the final 500 µg/mL treatment cocentration).
- 5000 µg/mL (diluted 1:2 in PBS; to achieve the final 1000 µg/mL treatment concentration).
- 10,000 µg/mL (used undiluted; to achieve the final 2000 µg/mL treatment concnetration).

To ensure sterility before inoculation, the reconstituted onion extract solutions were passed through a 0.22 μm syringe filter.

#### Treatment procedure

A hundred µL of each drug dilution was added to 400 µL of the *Blastocystis*-positive culture samples to achieve a final 1:5 volume ratio, the final ethanol concentrations in treatment wells were reduced to 0.2% (100 µg/mL treatment), 1.0% (500 µg/mL treatment), and 2.0% (1000 µg/mL treatment), which maintained solvent concentration ≤ 2%. The initial inoculum was standardized by adding a fixed amount of stool (50 mg) to a fixed volume of culture medium (50 ml) to maintain uniform starting conditions across all experimental arms. For each positive sample, the culture was divided equally into 10 untreated control tubes, which were maintained under identical conditions. Parasite counts were recorded daily over a 3-day incubation period, and. The average parasite counts of untreated samples served as the untreated control baseline against which drug efficacy was evaluated. The suspension was thoroughly homogenized before aliquoting into the separate treatment groups to assure homogeneity across all tubes at the beginning of the experiment. This was performed for all 21 biological samples included in the study. Separate culture groups prepared identically were treated with the respective test drugs. Counts were recorded daily over 3 days across all groups. A separate negative control (such as uninfected culture medium) was not included, as parasite reduction was directly compared against the parasite count in the untreated culture samples. The number of *Blastocystis* cells was counted using a hemocytometer under a light microscope every 24 h (x400) [25] and cell density was expressed as cells per milliliter (cells/mL). Viability was determined by eosin dye staining, where viable organisms remained unstained, and non-viable cells accepted the dye [26].

### GC-MS analytical protocols for GAPDH activity and no evaluation

#### Protein extraction and in vitro GAPDH reaction

*Blastocystis* culture samples were harvested by centrifugation at 2,000 × g for 10 min at 4 °C, washed three times with ice-cold PBS (pH 7.4), and lysed in chilled extraction buffer containing 50 mM Tris-HCl (pH 7.5), 1 mM EDTA, and 1% (v/v) protease inhibitor cocktail. Cell lysates were cleared by centrifugation at 12,000 × g for 15 min at 4 °C, and soluble protein concentrations were quantified via the Bradford assay.

For the GAPDH activity assay, normalized protein extracts (150 µg) were incubated at 37 °C for 30 min in a reaction mixture containing 100 mM Tris-HCl buffer (pH 7.5), 1 mM NAD⁺, 1 mM dithiothreitol (DTT), and 1 mM glyceraldehyde-3-phosphate (G3P) in a final volume of 200 µL. The enzymatic reaction was stopped on ice by adding 200 µL of ice-cold methanol to precipitate proteins. Following centrifugation at 12,000 × g for 10 min at 4 °C, the clear supernatant was collected and evaporated to complete dryness under a gentle stream of nitrogen gas.

#### Sample preparation and derivatization workflows

*GAPDH metabolite derivatization workflow*: Dried residues from the GAPDH reaction were resuspended in 20 µL of methoxyamine hydrochloride in pyridine (20 mg/mL) and incubated at 37 °C for 90 min to stabilize carbonyl groups. Silylation was subsequently performed by adding 80 µL of N, O-bis(trimethylsilyl)trifluoroacetamide (BSTFA) with 1% TMCS and incubating at 70 °C for 30 min to derivatize hydroxyl and phosphate groups of the glycolytic intermediates (G3P and 1,3-Bisphosphoglycerate (1,3-BPG)).

*NO chemical trapping workflow*: NO accumulation in culture supernatants was indirectly evaluated through chemical trapping. A volume of 200 µL of clarified culture supernatant was reacted with 50 µL of 2-phenyl-4,4,5,5-tetramethylimidazoline-1-oxyl 3-oxide (PTIO, 1 mM in PBS, pH 7.4) for 30 min at 25 °C in the dark. The resulting stable NO derivatives (NO-PTIO adducts) were extracted using 300 µL of ethyl acetate, dried over anhydrous sodium sulfate (Na2SO4), and concentrated under a nitrogen stream prior to GC-MS injection.

#### GC-MS instrumental settings and relative quantification

Chromatographic separation and mass detection were performed using a Shimadzu GCMS-QP2010 system (Shimadzu Corporation, Kyoto, Japan) coupled to a single quadrupole mass spectrometer. Separation was achieved using an Rtx-5MS capillary column (30 m × 0.25 mm i.d. × 0.25 μm film thickness; Restek, USA). Helium (99.999% purity) served as the carrier gas at a constant flow rate of 1 mL/minute. Samples (1 µL) were injected in splitless mode with an injection port temperature of 250 °C and an electron ionization (EI) source voltage of 70 eV.

*Oven temperature ramps*:

- For GAPDH Metabolites: The column oven temperature was initialized at 70 °C (held for 1 min), ramped at 10 °C/minute to 300 °C, and held for 5 min.
- For NO Derivatives: The column oven temperature was initialized at 40 °C (held for 1 min), ramped at 10 °C/minute to 250 °C, and held for 5 min.

Ion source and interface temperatures were maintained at 230 °C and 280 °C, respectively, for both methods.

*Detection, quality control, and quantification*: Mass spectrometry was operated in Selected Ion Monitoring (SIM) mode targeting specific characteristic ions.

*GAPDH assay targets*: m/z 319 and 361 for the G3P derivative; m/z 437 and 475 for the 1,3-BPG derivative.

*NO assay target*: m/z 30 and 156 for the NO derivative.

Pooled quality control samples and reagent blanks were analyzed every six runs to monitor column performance and instrument stability. Limits of detection (LOD) and quantification (LOQ) were defined as signal-to-noise ratios (S/N) of ≥ 3 and ≥ 10, respectively.

Due to the chemical instability and short half-life of intermediate glycolytic compounds (such as 1,3-BPG), relative quantification (metabolic profiling) was implemented by normalizing integrated target peak areas (m/z signals) to total protein content (mg protein). Instrumental response linearity and retention time reproducibility were monitored using serial dilutions of pooled quality control (QC) samples across analytical runs. Relative activity and metabolic levels were expressed as fold-changes relative to the untreated growth control.

### Statistical analysis

Statistical analysis was performed using Jamovi version 2.7.28. Assumption checks for normality and homogeneity of variances were performed by the Shapiro-wilk test and the Levene’s test, respectively. Since data violated normality assumptions, Kruskal-Wallis test was used for the analysis of quantitative data. Post hoc pairwise comparisons were conducted using the Dwass–Steel–Critchlow–Fligner (DSCF) test. Correlations between variables were evaluated using Spearman’s correlation coefficient. Statistical significance was set at *P* < 0.05.

For figure visualization, compact letter displays (a–f) were assigned to treatment groups based on DSCF post-hoc outcomes. Groups sharing the same letter represent no statistically significant difference, while different letters denote significant differences. The number of samples (*n* = 21) represents independent biological samples (with technical replicates averaged prior to analysis),

## Results

### Microscopic examination of study samples

**Fig. 1:**
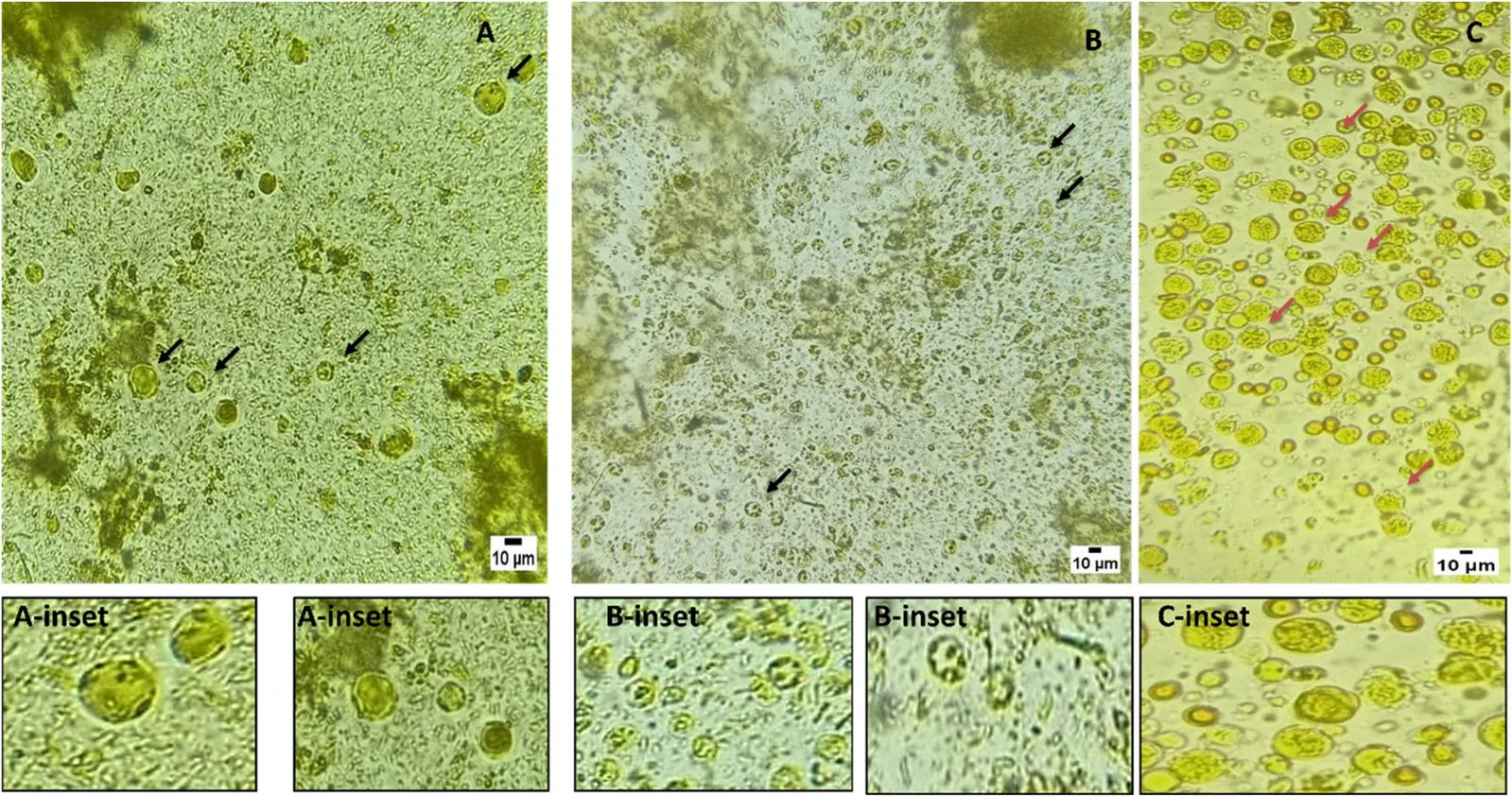
Photomicrographs showing different morphological forms of *Blastocystis* sp. observed in stool samples (400). **A**, **B** Vacuolar forms of *Blastocystis* sp. (indicated by black arrows) detected in wet mount preparations, characterized by a central vacuole surrounded by a thin peripheral cytoplasmic rim. **C** Granular forms of *Blastocystis* sp. (indicated by red arrows) detected in Jones’ culture medium, showing internal granules within the central vacuole and cytoplasm. Lower panels (**A**-inset, **B**-inset, and **C**-inset) provide close-up views for better visualization of structural features of the corresponding morphological forms above (Scale bars = 10 μm)

Microscopic examination of stool samples and cultured isolates revealed that the vacuolar and granular forms were the predominant morphological stages of *Blastocystis* sp. These forms were consistently observed in both direct wet mount preparations and cultured samples (Fig. 1).

### *Blastocystis* cell counts in cell culture

**Fig. 2:**
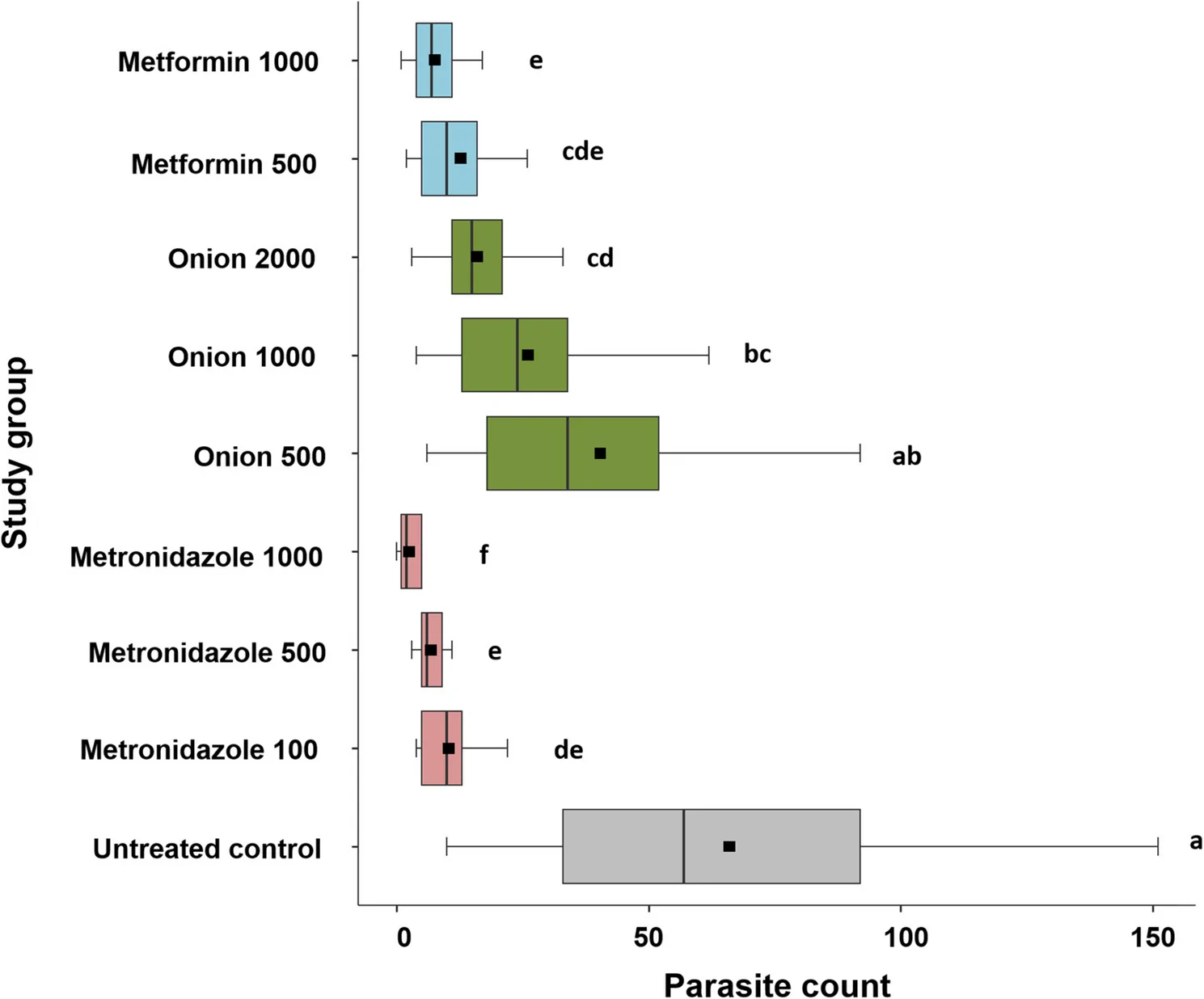
Boxplot showing means (black squares), medians and interquartile ranges (IQRs) of the parasite count (× 10⁴ cells/mL) in the various study groups; Drug concentrations in µg/mL; Color fills indicate treatment categories: untreated baseline control (gray), metronidazole (pink), onion extract (green), and metformin (blue). Groups labeled with different lowercase letters (a-f) are statistically significantly different from one another; *Statistical significance a *P* < 0.05 (Kruskal Wallis test followed by DSCF post hoc test)

The mean *Blastocystis* count in untreated cell culture was 66.0 ± 44.5 × 10⁴ cells/mL. All therapeutic agents, except for the onion 500 µg/mL preparation, significantly decreased the parasite count at different concentrations when compared to the control group. The higher the concentration of the drug, the more efficient it was in decreasing the count with some exceptions. Metronidazole was most efficient in decreasing *Blastocystis* count followed by metformin and onion preparations (Fig. 2).

### Nitric oxide levels and GAPDH activity in *Blastocystis* culture samples exposed to various test drugs

**Fig. 3:**
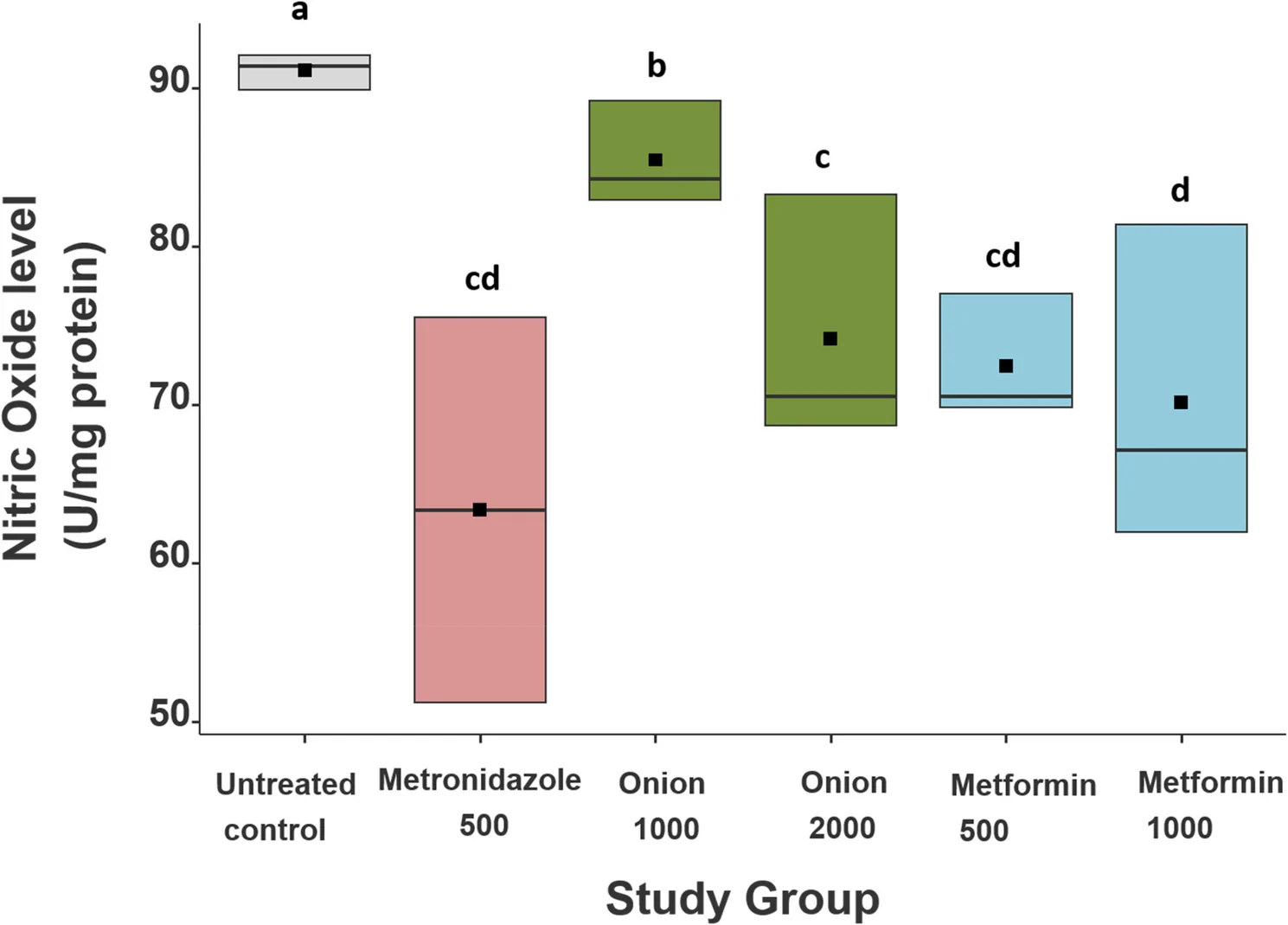
Boxplot showing means (black squares), medians and IQRs of NO levels in the various study groups. Drug concentrations in µg/mL; Color fills indicate treatment categories: untreated baseline control (gray), metronidazole (pink), onion extract (green), and metformin (blue). Groups labeled with different lowercase letters (a-d) are statistically significantly different from one another; *Statistical significance at *P* < 0.05 (Kruskal Wallis test followed by DSCF post hoc test)

**Fig. 4:**
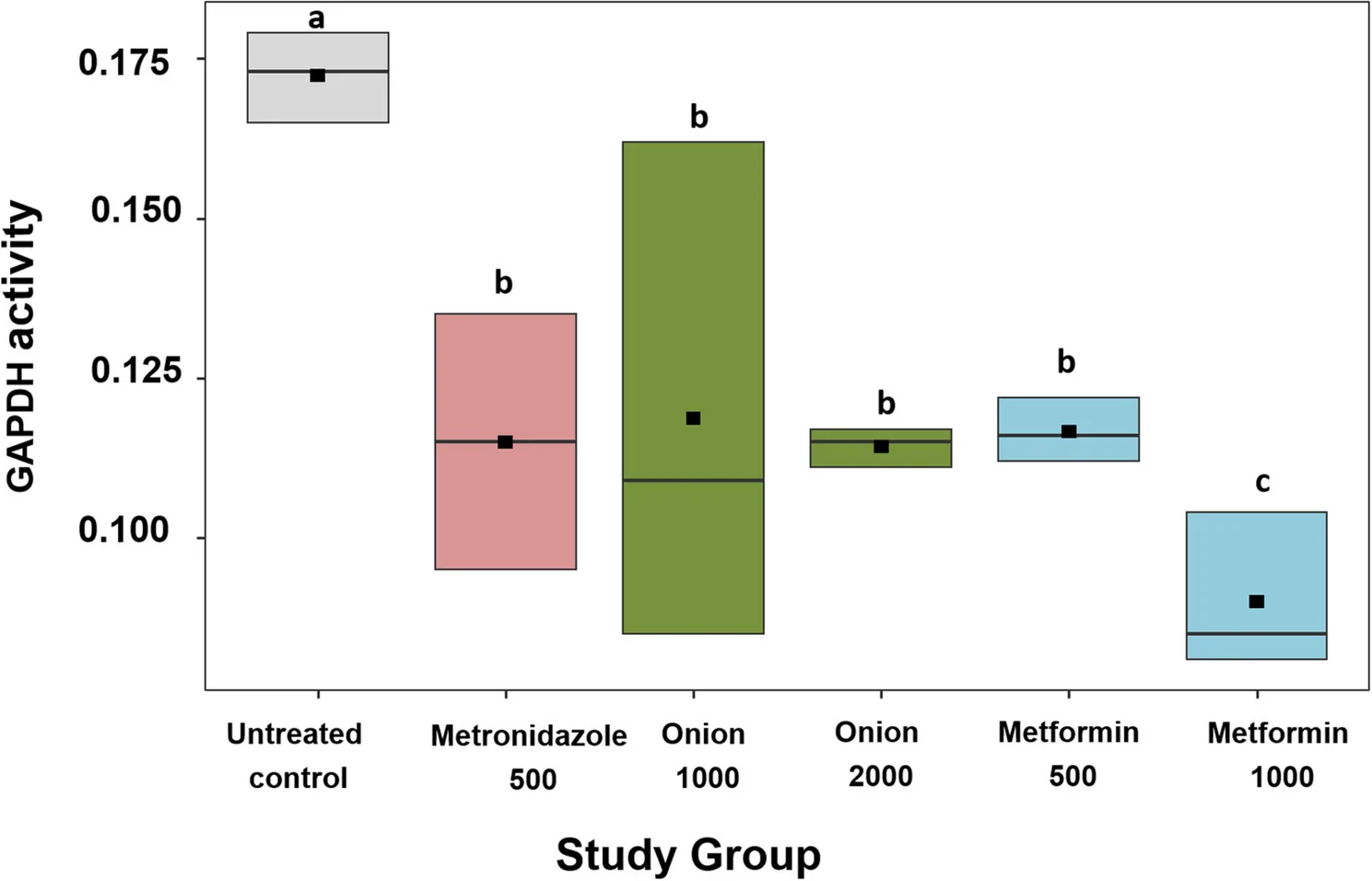
Boxplot showing means (black squares), medians and IQRs of GAPDH activity in the various study groups. Drug concentrations in µg/mL; Color fills indicate treatment categories: untreated baseline control (gray), metronidazole (pink), onion extract (green), and metformin (blue). Groups labeled with different lowercase letters (a-c) are statistically significantly different from one another; *Statistical significance at *P* < 0.05 (Kruskal Wallis test followed by DSCF post hoc test)

The highest NO level was observed in the untreated control group (91.1 ± 0.94 U/mg protein). *Blastocystis* culture samples exposed to the various therapeutic agents showed a significant reduction of NO levels, the lowest value being observed in the metronidazole 500 group (63.4 ± 12.14 U/mg protein) (Fig. 3). As for the GAPDH activity in cultured stool samples, the highest activity was detected in the untreated control group (0.17 ± 0.006). All tested agents showed a significant reduction in GAPDH activity in comparison to the untreated control group (Fig. 4). The lowest GAPDH activity was observed in the metfromin 1000 group (0.09 ± 0.010).

### Correlation between *Blastocystis* count, NO levels and GAPDH activity

Correlation between *Blastocystis* count, NO levels and GAPDH activity was analyzed by the Spearman correlation test. NO levels and GAPDH activity were found to be significantly negatively correlated in the metronidazole 500 group (*P* < 0.001), and significantly positively correlated in the metformin 1000 group (*P* < 0.001). Also, a significantly negative correlation was found between the *Blastocystis* count and the Onion 1000 group (*P* = 0.034) (Table 1).

**Table 1:** Correlation analysis between *Blastocystis* count, NO levels and GAPDH activity in the different study groups by Spearman ‘s correlation test

**Untreated control**		**Blastocystis** **count**	**Nitric Oxide level**	**GAPDH** **activity**
*Blastocystis* count	*Spearman’s Rho*	—		
	*P value*	—		
Nitric Oxide level	*Spearman’s Rho*	0.415	—	
	*P value*	0.185	—	
GAPDH activity	*Spearman’s Rho*	-0.039	0.500	—
	*P value*	1.000	0.063	—
***Metronidazole 500***		***Blastocystis*** **count**	**Nitric Oxide level**	**GAPDH** **activity**
*Blastocystis* count	*Spearman’s Rho*	—		
	*P value*	—		
Nitric Oxide level	*Spearman’s Rho*	0.190	—	
	*P value*	1.000	—	
GAPDH activity	*Spearman’s Rho*	-0.190	-1.000**	—
	*P value*	1.000	< 0.001	—
***Metformin 500***		***Blastocystis*** **count**	**Nitric Oxide level**	**GAPDH** **activity**
*Blastocystis* count	*Spearman’s Rho*	—		
	*P value*	—		
Nitric Oxide level	*Spearman’s Rho*	-0.490	—	
	*P value*	0.073	—	
GAPDH activity	*Spearman’s Rho*	0.412	-0.500	—
	*P value*	0.190	0.063	—
***Metformin 1000***		***Blastocystis*** **count**	**Nitric Oxide level**	**GAPDH** **activity**
*Blastocystis* count	*Spearman’s Rho*	—		
	*P value*	—		
Nitric Oxide level	*Spearman’s Rho*	-0.387	—	
	*P value*	0.249	—	
GAPDH activity	*Spearman’s Rho*	-0.387	1.000**	—
	*P value*	0.249	< 0.001	—
***Onion 1000***		***Blastocystis*** **count**	**Nitric Oxide level**	**GAPDH** **activity**
*Blastocystis* count	*Spearman’s Rho*	—		
	*P value*	—		
Nitric Oxide level	*Spearman’s Rho*	-0.540*	—	
	*P value*	0.034	—	
GAPDH activity	*Spearman’s Rho*	-0.415	0.500	—
	*P value*	0.185	0.063	—
***Onion 2000***		***Blastocystis*** **count**	**Nitric Oxide level**	**GAPDH** **activity**
*Blastocystis* count	*Spearman’s Rho*	—		
	*P value*	—		
Nitric Oxide level	*Spearman’s Rho*	-0.483	—	
	*P value*	0.080	—	
GAPDH activity	*Spearman’s Rho*	-0.068	0.500	—
	*P value*	1.000	0.063	—

Simultaneous multiple correlation comparisons using Bonferroni method

* *p* < 0.05, ** *p* < 0.001; Statistical significance at *P* < 0.05

## Discussion

The present study investigated the antiparasitic and biochemical effects of metronidazole, metformin, and onion extract on *Blastocystis* sp., with a focus on parasite burden, nitric oxide levels, and GAPDH activity within the microenvironment of the culture medium. The findings demonstrate that all tested agents significantly reduced *Blastocystis* and modulated key metabolic and nitrosative pathways in cultured samples, highlighting a potential therapeutic value against *Blastocystis*-associated GIT infection. To the best of our knowledge, this is the first study to simultaneously evaluate the effects of metronidazole, metformin, and *Allium cepa* extract on *Blastocystis* sp. culture through integrated assessment of parasite burden, GAPDH enzymatic activity, and NO levels using GC-MS-based analysis. This combined metabolic–nitrosative approach provides exploratory mechanistic insight into potential therapeutic targets beyond conventional parasite eradication.

NO plays a complex role in host–parasite interactions. While endogenous NO production has been demonstrated in several parasites, particularly helminths where it contributes to neuromuscular signaling and developmental processes, its presence in protozoa such as *Blastocystis* sp. remains uncertain [27]. To date, there is no clear evidence supporting the existence of NO synthase (NOS) in *Blastocystis*. Therefore, the NO detected in the present study is likely derived, at least in part, from co-existing microbiota within the xenic culture system, as previously suggested. The use of xenic culture represents a double-edged approach: while it limits the ability to attribute biochemical and metabolic changes exclusively to the parasite, it reflects the physiological intestinal microenvironment in which *Blastocystis* interacts with host microbiota. The bacterial community surrounding *Blastocystis* offers a syntrophic milieu for parasite growth by providing nutrients and consuming oxygen, which establishes the favorable anaerobic milieu required by *Blastocystis* sp. This explains the persistence of xenic culture media as indispensable tools for the in vitro propagation of *Blastocystis*, where axenic cultivation has succeeded in maintaining a restricted number of subtypes, excluding the predominant subtype 3 [28]. Jones’ culture medium is a monophasic liquid medium that continues to serve as the standard procedure for the propagation of *Blastocystis*. Although it selectively favors the growth of *Blastocystis* over bacteria, it remains to be a xenic culture medium that does not exclude the presence of gut bacteria [29]. Shaw et al. conducted microbiome and metabolome profiling among *Blastocystis* subtypes ST1-ST9 cultivated on modified Jones’ medium. The authors justified the use of this xenic culture medium for their work which presents a framework for deciphering subtype-specific biology by emphasizing the importance of the bacterial community in *Blastocystis* survival. In addition, the authors supported their choice by stating that subtypes such as subtype 3, one of the most prevalent *Blastocystis* subtypes, cannot be successfully cultivated on axenic culture media [28].

Importantly, NO has been identified as a key effector molecule against *Blastocystis* sp. Mirza et al. [10] demonstrated that subtypes 4 and 7 are susceptible to NO donors, with subtype 7 showing increased sensitivity. Notably, subtype 7 was also capable of downregulating inducible NOS (iNOS) in intestinal epithelial cells, suggesting an adaptive immune evasion mechanism. In the same context, the generation of NO in cultured *Blastocystis* samples observed in our study may reflect the host response to infection by inducing a state of oxidative stress. NO level reduction in treated samples may also result from the modulation of the associated microbial community, highlighting the complexity of the nitrosative response to *Blastocystis* co-infection within the GIT.

Metronidazole exhibited the most pronounced reduction in parasite count, consistent with its established role as a first-line anti-*Blastocystis* agent. Its mechanism involves the generation of reactive nitrogen species that induce DNA damage and apoptosis-like cell death in anaerobic organisms [30, 31]. Interestingly, despite its nitrosative mode of action, metronidazole treatment in this study was associated with reduced NO levels. This paradox may be explained by its antimicrobial effects on co-existing bacteria in xenic cultures, which likely contribute to NO production. Metronidazole was also found to reduce GAPDH activity in *Blastocystis* culture. GAPDH is a catalytic enzyme that converts glyceraldehyde triphosphate into1,3-BPG. In addition to its role in glycolysis, it exerts important cellular functions such as the response against oxidative stress, the protection and preservation of DNA structure, and apoptosis. These functions are under a complex regulatory machinery, one of them being post-translational modification. Nitric oxide was found to modify GAPDH not only by oxidation, but also by ribosylation and nitrosylation [9, 32].

GAPDH was found to be produced by parasites like *Leishmania major*, where it inhibits the production of TNF-α in infected macrophages [33]. GAPDH has been identified as a part of a fusion protein with TPI in *Blastocystis hominis*. Glycolytic enzymes constitute potential drug targets in the management of infectious agents [2]. Motlhatlhedi et al. [8] reported the in-silico discovery of anti-schistosomal small molecules that target the GAPDH-TPI fusion protein resulting in the dual inhibition of both enzymes.

The significant inhibition of GAPDH activity by metformin, particularly at higher concentrations, suggests a strong metabolic effect that may impair parasite survival. Metformin demonstrated notable antiparasitic activity, supporting previous studies on its immunomodulatory and antimicrobial properties. In a study conducted by Salama et al. [14], metformin was found to have a potent synergistic effect to albendazole against experimental *Toxocara canis* infection. The drug combination significantly decreased larval burden and improved organ pathology. Metformin and albendazole also decreased oxidative stress, as evidenced by decreased malondialdehyde and NO levels, and increased superoxide dismutase and catalase activity. Jauvain et al. [34], reported that metformin reduced the relative abundance of the intestinal microbiota in *Helicobacter pylori (H. pylori)* -infected mice. They also conducted a computational analysis which revealed a functional effect of metformin on the nitrate and nitrite reducing abilities of bacteria. N-nitroso compounds produced by gut microbes are recognized promoters of gastrointestinal mutagenesis. Metformin was also found to attenuate the *H. pylori*-induced increase in microbial carbohydrate metabolism. One of the products of carbohydrate metabolism in bacteria are short chain fatty acids, which are also implicated in intestinal carcinogenesis. Moreover, the positive correlation between NO levels and GAPDH activity in metformin-treated cultures suggests a complex interaction between metabolic and nitrosative pathways that warrants further investigation.

Onion has been evaluated as an antiparasitic agent in several studies. Orengo et al. [35] conducted an in vitro study on the effect of onion and other herbal extracts on *Toxocara canis* and *Ancylostoma caninum*. Onion was efficient in inhibiting egg hatching and propagation into larval stages. Krstin et al. [36] reported that onion shows anti-trypanosomal and anti-leishmanial effects. Kim et al. [37] demonstrated that onion exerts antioxidant and anti-inflammatory activities, where the exposure of mouse-derived RAW 264.7 macrophages to whole onion extracts resulted in the upregulation of antioxidant enzymes such as superoxide dismutase and catalase, and the decreased production of NO. In addition, whole onion extracts led to the downregulation of pro-inflammatory cytokines such as TNF-α and IL-6 in exposed macrophages. In the current study, onion extract also exhibited antiparasitic effects, although less pronounced than metronidazole and metformin. Its activity may be attributed to bioactive compounds such as quercetin and organosulfur constituents, which exert antioxidant and enzyme-modulating effects [38]. The delayed effect observed in this study aligns with previous findings indicating time-dependent antiparasitic activity of herbal extracts [15].

## Conclusion, limitations and recommendations

The current study demonstrates significant in vitro anti-*Blastocystis* activities exerted by metronidazole, metformin, and onion extract (*Allium cepa*), as evidenced by the obvious reduction in parasite burden. Regarding their biochemical and metabolic effects on the stool culture environment, the tested agents induced a decrease in NO levels and GAPDH activity. While metronidazole demonstrated the strongest direct antiparasitic effect, metformin-treated cultures exhibited the most pronounced suppression of total GAPDH metabolic activity, highlighting its potential as a metabolic-targeting agent within the culture ecosystem. These findings support the strategic value of targeting oxidative and glycolytic pathways as a novel therapeutic avenue against *Blastocystis*-associated intestinal inflammation within the holobiotic frame of microbial co-colonization. The demonstrated metabolic effects of these drugs underscore the value of therapeutic repurposing of pharmacological agents and the rediscovery of natural plant remedies as potential strategies against *Blastocystis*-associated intestinal infection.

A key limitation of this study is the reliance on xenic culture conditions without a parallel, *Blastocystis*-free treated stool control. Jones’ medium is an efficient culture medium that provides selective support for parasite multiplication, therefore allowing a better evaluation of the tested agents on parasite count. In addition, the use of xenic culture media maintains the realistic poly-microbial environment of the human gut, thereby reflecting a collective microenvironmental response rather than isolated, parasite-specific mechanisms. It must therefore be acknowledged that the observed metabolic and nitrosative suppression cannot be attributed to an isolated effect on *Blastocystis* viability but may also be emphasized by changes in the microbial milieu in which the growth of *Blastocystis* sp. has been augmented. Hence, this xenic framework provides a bio-mimetic representation of the intestinal environment in which the parasite naturally resides and provides a framework for future research specifically targeting *Blastocystis*-induced immunopathology. This approach may offer a translational bridge by repurposing pharmacological and herbal agents that modulate the local intestinal metabolic and oxidative status. In addition, the choice of drug concentrations for our experimental design was particularly challenging, due to the scarcity of literature reports on the in vitro effects of metformin and onion extracts on *Blastocystis*, especially when studied with a biochemical or metabolic framework. Doses were determined based on pilot trials and previous studies on in vitro applications of the drugs. Since the study was designed to serve as a preliminary exploratory investigation of potential anti-*Blastocystis* and metabolic drug effects, we opted to use fixed drug concentrations instead of determining IC₅₀/EC₅₀ and calculating full dose response curves. Nevertheless, the validation of the effects of these drugs on *Blastocystis* and related metabolic and biochemical changes by pharmacodynamic and pharmacokinetic approaches is recommended to advance to the translational application of these drugs against blastocystosis. In addition, the lack of vehicle controls and negative bacterial controls is an important limitation that must be acknowledged. The different solvent concentrations in the different drug preparations after dilution complicates the preparation of unified vehicle control. Nevertheless, several measures such as filtration, rotary evaporation and dilution with sterile physiological PBS were done to maintain solvent concentrations below levels that can produce a functional effect. Although both GADPH activity and NO levels showed a significant reduction in treated culture samples showing significant reduction of parasite count as compared to the untreated positive control, without multi-omics approaches causal conclusions cannot be drawn. Future investigations utilizing axenic cultures or comparative *Blastocystis*-negative fecal controls are warranted to identify specific molecular pathways implicated in the pathogenesis of *Blastocystis*-associated GIT infections. Additionally, further research exploring using multi-omics approaches on how *Blastocystis* interacts with and modulates environmental nitrosative stress will provide essential insights into parasite survival mechanisms and microbiome-mediated therapeutic targeting.

## Supplementary Information

Supplementary Material 1.

## Data Availability

The data used and analyzed during the current study are available from the corresponding author upon reasonable request.

## Abbreviations

DTT: Dithiothreitol
1,3-BPG: 1,3-Bisphosphoglycerate
BSTFA: N, O-bis(trimethylsilyl)trifluoroacetamide
DSCF test: Dwass–Steel–Critchlow–Fligner test
EI: Electronic ionization
G3P: Glyceraldehyde-3-phosphate
GAPDH: Glyceraldehyde-3-phosphate dehydrogenase
GC-MS: Gas Chromatography–Mass Spectrometry
H. pylori: Helicobacter pylori
iNOS: inducible nitric oxide synthase
IQR: Interquartile range
LOD: Limits of detection
LOQ: Limits of qauntification
NO: Nitric oxide
NOS: Nitric oxide synthase
NSAIDs: Non-steroidal anti-inflammatory drugs
PBS: Phosphate-buffered saline
PTIO: 2-phenyl-4,4,5,5-tetramethylimidazoline-1-oxyl 3-oxide
QC: Quality control
SIM: Selected Ion Monitoring
TPI: Triosephosphate isomerase
