# Supplementary material for: Evaluating the effects of metronidazole, metformin, and onion extract on GAPDH activity and nitric oxide production in Blastocystis sp. cultures: a biochemical and therapeutic approach
Source: BMC Complement Med Ther. 2026 Oct 12;26:295. doi: 10.1186/s12906-026-05605-x (PMC13656695; doi:10.1186/s12906-026-05605-x)
Supplement: Supplementary file 1 [file 12906_2026_5605_MOESM1_ESM.docx]

**Supplementary file**

Manuscript title: **Metabolic and Nitrosative Targeting of *Blastocystis* sp.: Comparative In Vitro Effects of Metronidazole, Metformin, and *Allium cepa* Extract**

**Table 1:** Non-parametric One-Way ANOVA (Kruskal-Wallis) test and effect sizes across evaluated parameters of *Blastocystis* sp. following treatment.

| ***Study parameters*** | χ² | df | p | ε² |
| --- | --- | --- | --- | --- |
| Parasite count | 117 | 8 | <.001 | 0.621 |
| Nitric Oxide level | 88.8 | 5 | <.001 | 0.711 |
| GAPDH activity | 79.4 | 5 | <.001 | 0.635 |

**Table 2:** Between group comparison between various study groups using the DSCF post hoc test

| ***Pairwise comparisons (DSCF test)- Parasite count*** | | **P-value** |
| --- | --- | --- |
| Untreated control | Metronidazole 100 | <.001^*^ |
| Untreated control | Metronidazole 500 | <.001^*^ |
| Untreated control | Metronidazole 1000 | <.001^*^ |
| Untreated control | Metformin 500 | <.001^*^ |
| Untreated control | Metformin 1000 | <.001^*^ |
| Untreated control | Onion 500 | 0.658 |
| Untreated control | Onion 1000 | 0.024^*^ |
| Untreated control | Onion 2000 | <.001^*^ |
| Metronidazole 100 | Metronidazole 500 | 0.496 |
| Metronidazole 100 | Metronidazole 1000 | <.001^*^ |
| Metronidazole 100 | Metformin 500 | 0.999 |
| Metronidazole 100 | Metformin 1000 | 0.808 |
| Metronidazole 100 | Onion 500 | <.001^*^ |
| Metronidazole 100 | Onion 1000 | 0.020^*^ |
| Metronidazole 100 | Onion 2000 | 0.384 |
| Metronidazole 500 | Metronidazole 1000 | <.001^*^ |
| Metronidazole 500 | Metformin 500 | 0.295 |
| Metronidazole 500 | Metformin 1000 | 1.000 |
| Metronidazole 500 | Onion 500 | <.001^*^ |
| Metronidazole 500 | Onion 1000 | <.001^*^ |
| Metronidazole 500 | Onion 2000 | 0.007^*^ |
| Metronidazole 1000 | Metformin 500 | <.001^*^ |
| Metronidazole 1000 | Metformin 1000 | 0.005^*^ |
| Metronidazole 1000 | Onion 500 | <.001^*^ |
| Metronidazole 1000 | Onion 1000 | <.001^*^ |
| Metronidazole 1000 | Onion 2000 | <.001^*^ |
| Metformin 500 | Metformin 1000 | 0.573 |
| Metformin 500 | Onion 500 | 0.001^*^ |
| Metformin 500 | Onion 1000 | 0.109 |
| Metformin 500 | Onion 2000 | 0.844 |
| Metformin 1000 | Onion 500 | <.001^*^ |
| Metformin 1000 | Onion 1000 | 0.001^*^ |
| Metformin 1000 | Onion 2000 | 0.029^*^ |
| Onion 500 | Onion 1000 | 0.763 |
| Onion 500 | Onion 2000 | 0.033^*^ |
| Onion 1000 | Onion 2000 | 0.665 |
| **Statistical significance at P<0.05* | | |

Table 3: Pairwise post hoc comparison between NO levels and GAPDH levels in the various study groups

| *Pairwise comparisons – P values for DSCF test; Statistical significance at P<0.05* | | | |
| --- | --- | --- | --- |
| **Study groups** | | **GAPDH**  **activity** | **NO level** |
| Untreated control | Metronidazole 500 | <.001^*^ | <.001^*^ |
| Untreated control | Metformin 500 | <.001^*^ | <.001^*^ |
| Untreated control | Metformin 1000 | <.001^*^ | <.001^*^ |
| Untreated control | Onion 1000 | <.001^*^ | <.001^*^ |
| Untreated control | Onion 2000 | <.001^*^ | <.001^*^ |
| Metronidazole 500 | Metformin 500 | 1.000 | 0.305 |
| Metronidazole 500 | Metformin 1000 | 0.002^*^ | 0.410 |
| Metronidazole 500 | Onion 1000 | 1.000 | <.001^*^ |
| Metronidazole 500 | Onion 2000 | 1.000 | 0.305 |
| Metformin 500 | Metformin 1000 | <.001^*^ | 0.418 |
| Metformin 500 | Onion 1000 | 0.418 | <.001^*^ |
| Metformin 500 | Onion 2000 | 0.418 | 0.989 |
| Metformin 1000 | Onion 1000 | 0.002^*^ | <.001^*^ |
| Metformin 1000 | Onion 2000 | <.001^*^ | 0.022^*^ |
| Onion 1000 | Onion 2000 | 0.418 | <.001^*^ |
| *Statistical significance at P<0.05 | | | |
